# Parochial Lunacy Reform in Scotland

**Published:** 1862-04

**Authors:** 


### Art. VIII.—PAROCHIAL LUNACY REFORM IN SCOTLAND.

For several months back, an agitation has been in progress among certain of the parochial representatives of Scotland, for an amendment of the Lunacy Act of that part of the kingdom. This Act, rendered necessary by the results of an inquiry, conducted by a Royal Commission, into the condition and provision for the care of the insane in Scotland, received the Royal Assent so recently as the 25th of August, 1857, and its chief objects were to provide for the building of district asylums for the reception of pauper lunatics, and to ensure the proper care and treatment of lunatics generally, whether placed in asylums, or left in private houses under the care of relatives or strangers. A Board constituted of five Commissioners, aided by two Deputy or Assistant-Commissioners, was appointed, according to the provisions of the Act, to carry its requirements into effect; and it is but an act of the simplest justice to add that the Board and the Assistant-Commissioners, justly appreciating the true spirit of the statute, have fulfilled the onerous and delicate duties entrusted to them in a manner worthy of the highest approbation. The powers of the Board of Commissioners will cease in January, 1863, and it is provided that thenceforward these powers shall be vested in the two present paid Commissioners, to be termed Inspectors-General of Lunacy, and who shall be subject only to the Secretary of State.

The leading features of the agitation at present going on for the amendment of the existing Lunacy Act, would appear to be, so far as yet developed, as follows:—

1. To draw a distinction between private and pauper insanity, and to place the care and treatment of the former under the direction of the Commissioners in Lunacy: while the powers of the Board of Lunacy, so far as they relate to *pauper* lunatics, are to be transferred to the Board of Supervision for relief of the poor.

2. The recognition of accommodation for pauper lunatics under the title of "Parochial Asylums," being either part of, or in connexion with the poorhouses.

3. The suppression of the visitation of patients residing in their own homes, as at present conducted, by the Commissioners and Deputy-Commissioners.

By the change first proposed is involved a return to the vicious system of management followed up to 1857, the evil results of which were fully shown in the Report of the Royal Commission of 1857. By this change many of the humane characteristics which distinguish the existing Act would be altogether done away with; for the result would be to associate the management of lunacy with that of pauperism, and to place the care and treatment of the former in the hands of Parochial Boards, who, from past experience, have shown themselves far from ready to recognise the claims of insanity on their consideration. It would be easy to multiply examples of the evils which flow from the management of the insane being placed in the hands of Parochial Boards. Their powers, indeed, are in many cases delegated to the Inspector of Poor, who would, under the system now proposed, have the control and direction of the insane poor belonging to the parish.

Against the second proposal the greater part of the ratepayers of Scotland may be said to have declared themselves, for they have recognised the existing Act to such an extent that, where asylums were not already within the district, they have admitted the necessity for them by procuring ground, obtaining plans, and making arrangements which leave no reason to doubt that, were the present Act to continue in force, asylums in the following districts would ere long be erected.

| | | |
|---|---|---|
| Argyll. | Inverness. | Stirling. |
| Banff. | Ross. | Linlithgow. |
| Fife. } | Sutherland. } | Dumbarton. } |
| Kinross. } | Nairn. } | Clackmannan. } |
| Haddington. | Perth. | |

Asylums exist in the following districts:—

| | | |
|---|---|---|
| Aberdeen. | Edinburgh. } | Lanark. |
| Dumfries. } | Peebles. } | Elgin. |
| Kirkcudbright. } | Forfar. } | |
| Wigtown. } | Kincardine. } | |

Against the twenty-four counties here represented, we place the following as those in which the provisions of the Act have not been acted upon in respect to the provision of district asylums.

| | | |
|---|---|---|
| Ayr. | Orkney. | Roxburgh. } |
| Caithness. | Shetland. | Berwick. } |
| Bute. | Renfrew. | Selkirk. } |

With the exception of Ayr, Renfrew, and the three last counties in association, the erection of separate asylums has not been contemplated, for it was presumed that such counties as Caithness, Bute, Orkney, and Shetland, would enter into contracts with other districts for the reception of their pauper lunatics. Indeed, in the largest of these counties, Caithness, negotiations were at one time in progress with the district of Inverness for admission into that combination. The proposal now made therefore can have reference only to Ayr, Renfrew, Roxburgh, Berwick, and Selkirk; and here, it is true, parochial asylums may be erected, but why the insane poor of these counties should be deprived of the care bestowed upon the rest of Scotland is not apparent.

The third proposition may be said perhaps to be the most harsh in its tendency. It is to declare that the misery and wretchedness which exist in the private dwellings of the insane, as largely set forth in the Reports of the Lunacy Commissioners, are matters with which the Legislature may not deal, even in the way of supervision. It is to declare that cases of neglect and suffering, of bad accommodation and insufficient allowance, of injudicious management and want of surveillance, and of erotic imbeciles at large, such as those brought to light in the first Report of the Board of Lunacy,* may continue in the same state of degradation, uncared for. For as we know that many such cases continue to exist, we cannot doubt that when the influence of visitations such as those instituted by the Board of Lunacy is lost, the condition of those lunatics who have already, thanks to the existing statute, been rescued from wretchedness and placed in a comparatively favourable position, will ere long fall back into the same pitiable state as when the Commissioners' visitations began.

It is significant that the proposals now adverted to have emanated from certain Inspectors of Poor, who have assumed to themselves the duty of legislating for the insane of Scotland generally, and we find, accordingly, that the whole spirit of the movement is one of reducing to the lowest amount possible the care and pecuniary outlay extended to pauper lunatics. In effect it is to provide a covering, and perhaps sufficient food for these unfortunates, but to place out of view all those higher considerations, not to say duties, which prompt us to claim for them increased care and sympathy.

We have such faith in the sound feeling of our northern brethren as to believe that an agitation so entirely subversive of the more enlightened principles which have happily for several years governed lunacy legislation in the United Kingdom, must entirely fail in attaining its objects.

[The preceding remarks were already in type, when we were given to understand that the contemplated Act embodying the proposed changes had been withdrawn.]

---

* See the numerous cases recorded, pp. 176—220.

